# Inhibition of GSK3β activity alleviates acute liver failure via suppressing multiple programmed cell death

**DOI:** 10.1186/s12950-023-00350-1

**Published:** 2023-07-13

**Authors:** Danmei Zhang, Chunxia Shi, Qingqi Zhang, Yukun Wang, Jin Guo, Zuojiong Gong

**Affiliations:** Department of Infectious Diseases, Renmin Hospital of Wuhan University, No. 238 Jiefang Road, Wuhan, Hubei province 430060 China

**Keywords:** Acute liver failure, GSK3β, TAK1, TRAF6, HDAC3, Programmed cell death

## Abstract

**Background:**

Acute liver failure (ALF) is one of the most common life-threatening diseases in adults without previous liver disease. Glycogen synthase kinase 3β (GSK3β) is a serine/threonine protein kinase that is widely distributed in the cells. Inhibition of its activity can inhibit cell death and promote autophagy through various pathways, thus providing a protective effect. In this study, we aimed to investigate the effect on ALF after inhibition of GSK3β and its potential mechanisms.

**Methods:**

D- galactosamine(D-Gal) in combination with lipopolysaccharide(LPS) was used to induce ALF in vitro and in vivo. And then GSK3β inhibitor TDZD-8 was used to explore the protective effect against ALF. After TDZD-8 treatment TUNEL staining and flow techniques were used to detect the proportion of apoptosis in liver tissues and cells respectively, while western blotting and immunofluorescence assays were performed to detect the expression levels of apoptosis, pyroptosis and necroptosis-related proteins in tissues and cells. In addition, western blotting was performed to explore the specific mechanism of hepatoprotective effect after GSK3β inhibition to detect the expression levels of TAK1, TRAF6 and HDAC3 after TRAF6 and HDAC3 inhibition alone. The co-localization of TRAF6 and HDAC3 in vitro was detected by immunofluorescence, while the interaction between TRAF6 and HDAC3 was detected by immunoprecipitation assay.

**Results:**

Both in vivo and in vitro experiments, GSK3β inhibitor TDZD-8 can significantly alleviate the progression of ALF. Inhibition of GSK3β activity could significantly reduce the level of hepatocyte apoptosis, pyroptosis, necroptosis and improve liver dysfunction and tissue damage. Furthermore, we found that hepatocyte TAK1 and TRAF6 levels decreased and HDAC3 levels increased in ALF, whereas inhibition of GSK3β upregulated TAK1 and TRAF6 levels and decreased HDAC3 expression.

**Conclusion:**

GSK3β inhibitor TDZD-8 can prevent the progression of ALF, and its action may involve the TRAF6/HDAC3/TAK1 pathway.

**Supplementary Information:**

The online version contains supplementary material available at 10.1186/s12950-023-00350-1.

## Introduction

Acute liver failure(ALF) is a serious clinical syndrome leading to massive liver cell death in a short time, usually caused by various factors, including drugs, toxins, viruses, and genetic disorders, without a history of liver disease [1]. Patients with ALF have a very high mortality rate and poor prognosis if not treated in time. To date, for these patients, there is a lack of effective drugs and treatments other than liver transplantation. Therefore, finding new therapeutic targets and developing new treatment approaches are still under evaluation.

Animal models formed by intraperitoneal injection of lipopolysaccharide (LPS) and D-galactosamine (D-Gal) are widely used in human ALF pathogenesis and drug development because their clinical signs are similar to those of patients [2]. Excessive inflammatory response and massive hepatocyte death are the main features of ALF [3]. In the available studies, the LPS/D-Gal model exhibits typical hepatocyte death, including apoptosis, necroptosis, autophagy and other programmed deaths. In addition, although not well studied, hepatocyte death caused by pyroptosis and necroptosis in the LPS/D-Gal model has been reported to be associated with severe liver injury [4]. Thus, multiple modes of death may co-exist in the ALF model, collectively leading to liver damage and poor prognosis.

Glycogen synthase kinase 3β (GSK3β) is a serine/threonine protein kinase prevalent in a variety of cells and is originally thought to be involved in cellular metabolism, growth and other physiological functions [5]. With progressive research, more and more studies have concluded that GSK3β is associated with the occurrence of various diseases such as neuroinflammatory diseases, autoimmune diseases, inflammatory diseases, etc. [6, 7]. It is involved in various signaling pathways such as cellular inflammation, oxidative stress and apoptosis, and is considered to be a key regulatory molecule of the inflammatory response [8]. In addition to regulating inflammation, GSK3β is also implicated in cell death. For example, in one study GSK3β was identified as a key regulator of necrotic death [9], and selective inhibition of GSK3β was able to decrease necrosis-associated protein expression, reduce its interaction with receptor-interacting protein kinase 1 (RIPK1) and prevent ischemic stroke-induced scar formation [10]. In an acute liver failure model, GSK3β, which is highly activated, can aggravate liver inflammation and hepatocyte apoptosis in model mice and promote the progression of injury [11]; hepatoprotective drugs can improve hepatocyte apoptosis and iron death through GSK3β/Nrf2, AKT/GSK3β and other pathways and play a hepatoprotective role [12].

Programmed cell death mainly consists of apoptosis, necroptosis and pyroptosis. Previous studies have suggested that these three types of death exist independently and do not interfere with each other. Today, a growing number of studies suggest that these three types of programmed death can be activated simultaneously and affect each other in crosstalk in response to stimuli such as infection, Interferon regulatory factor 1 (IRF1) and tumor necrosis factor-α (TNF-α), a phenomenon known as PANoptosis [13]. Transforming growth factor beta-activated kinase 1 (TAK1) plays an important function in body immunity, participating in cellular pro-survival signal pathways and, when inactivated or absent, is usually able to initiate cellular PANoptosis [14]. Studies at this stage show that PANoptosis is involved in tumorigenesis, microbial infections, and auto-inflammatory diseases [15]. Oxidative stress activates PANoptosis in colon cancer cells, and to a certain extent alleviates the drug resistance of tumor cells. In infectious diseases, pathogens produce various inflammatory factors, which activate PANoptosis, so in terms of molecular mechanisms, PANoptosis can also be considered at the center of a cytokine storm [16]. The role of PANoptosis in ALF has not been studied enough, but ALF models harbor severe inflammatory responses, apoptosis, necrosis and scorching at the same time. In addition, GSK3β inhibitors can play a role in organ protection by alleviating oxidative stress and improving apoptosis. For this reason, we hypothesized whether GSK3β inhibitors could serve a hepatoprotective function and improve hepatic failure by inhibiting multiple modes of cell death in ALF.

Here, we have explored the protective effect of GSK3β inhibitor TDZD-8 against liver death induced by D-Gal/LPS in vivo and in vitro. Deeply, we also explored how inhibition of GSK3β ameliorates hepatocyte death while potential protein mechanisms are involved, providing new directions for the treatment of liver failure.

## Materials and methods

### Animal and experimental model

Male C57BL/6 mice (age, 7–8 weeks), which were provided by the Experimental Animal Center of Wuhan University, were housed in a specific pathogen-free facility at the animal experiment center in Renmin Hospital of Wuhan University with a light-controlled, the temperature of 25 ℃ and humidity of 50% ± 15%. All the animals can eat and drink freely. This project was approved by the Institutional Animal Care and Use Committee of Renmin Hospital of Wuhan University. All mice were randomly divided into three treatment groups: saline control group (*n* = 6); ALF model group (*n* = 6); ALF + TDZD-8 group (*n* = 6). All mice were injected intraperitoneally with D-Cal (400 mg/kg, Sigma-Aldrich, USA) and LPS (100 μg/kg, Sigma-Aldrich, USA), except for the control group. The control group was given equal amounts of saline. In addition, mice in the TDZD-8 group were treated with the GSK3β inhibitor TDZD-8 (2 mg/kg, MCE, USA) intraperitoneally for 2 h before the construction of the ALF model. The rest of the mice were injected with equal amounts of saline. The mice were treated 24 h after the construction of the ALF model, and the livers and serum were collected.

### Cell culture

The mouse normal hepatocyte line AML-12 was purchased from the Cell Collection Center of Wuhan University (Wuhan, China). The cells were incubated in Dulbecco^’^s Modified Eagle^’^s medium (DMEM/F12) (HyClone, USA) supplemented with 10% fetal bovine serum(FBS) (GIBCO, USA) at the 37 ℃, 5% CO_2_ concentration and saturated humidity environment. LPS (100 ng/mL, Sigma-Aldrich, USA) combined with D-Gal (44 μg/mL) treated cells were used to construct an in vitro model of acute hepatocyte injury, stimulating all cells except the control group. TDZD-8 (20 μM) and RGFP966 (15 μM, MCE, USA) were added after 24 h of cell culture. TDZD-8 is an inhibitor of GSK3β, which increases serine 9 (Ser 9) of GSK3β and inhibits the activity of this molecule; RGFP966 is a specific inhibitor of histone deacetylase 3 (HDAC3) and does not affect other HDAC molecules at concentrations of 15 μM and above, and is often used in experimental studies to inhibit HDAC3 activity [17].

### Small Interfering RNA (siRNA) transfection

Cells were seeded in 6-well plates at 50,000 cells per well to enable cells to reach a density of 70–80% by the next day. siRNA (RiboBio, Guangzhou, China) was used to transfect the si-(tumor necrosis factor receptor–associated factor 6) TRAF6 and si-TRAF6 + TDZD-8 groups. Meanwhile, non-targeted siRNA transfection of other groups was used as a negative control. Cells were treated with therapeutic intervention and molding 24 h after transfection, and cells were collected for protein concentration assay after 24 h of continued culture.

### Histological analysis and biochemical tests

Fresh liver tissue from mice was fixed in 10% neutral buffered formalin and paraffin-embedded sections were made after 24 h. Each mouse liver section was stained for HE and the histopathological changes were observed by microscopy. Blood from mice was collected by centrifugation at 3500 rpm for 15 min and the supernatant was aspirated. Serum levels of alanine aminotransferase (ALT) and aspartate aminotransferase (AST) were altered using a fully automated biochemical analyzer.

### TUNEL staining

Terminal deoxynucleotidyl transferase-mediated end-labeling assay was used to detect apoptosis in the tissues. Paraffin sections of liver tissue were dewaxed and hydrated, and proteinase K (Roche, USA) was added for 20 min at room temperature, followed by 3 washes in PBS. The TUNEL assay was added dropwise according to the instructions of the one-step TUNEL Apoptosis Assay Kit (Beyotime, Shanghai, China) and incubated for 1 h at 37 °C in a dark humidified chamber, followed by 3 washes with PBS. The nuclei were stained with DAPI and washed 3 times with PBS after 5 min. The slices were blocked using an anti-fluorescence quencher and the level of apoptosis in the liver tissue was observed under a fluorescent microscope.

### Flow cytometry

The Annexin V-PE/7-ADD Apoptosis Kit (BD, USA) was applied to detect the level of apoptosis in the different intervention cells. Cells were inoculated in 6-well plates at a density of 1 × 10^5^ per well, collected after stimulation by drug intervention, washed in PBS, and resuspended in 400 μL of buffer, 5 μL Annexin V-PE and 5 μL 7-AAD were added sequentially, incubated for 15 min at room temperature and protected from light, and then assayed for apoptosis using CytoFLEX (Beckman Coulter Biotechnology (Suzhou), China) to detect apoptotic cells.

### Immunofluorescence

Cells were seeded in 24-well plates containing circular slides and fixed in 4% paraformaldehyde for 25 min; for tissues, paraffin sections were dewaxed and hydrated, and repaired using sodium citrate antigen. After PBS washing, BSA was used to block at room temperature for 1 h. Rabbit anti-TAK1 (1:100, Abcam, ab10952), HDAC3 (1:100, Abcam, ab32369), RIPK1 (1.00, Proteintech, 17,513–1-AP), Caspase-8 (1:100, Abcam, ab25901) and mouse anti-TRAF6 (1:10, Santa, sc-8409) polyclonal antibodies were incubated overnight at 4 °C. The next day, secondary antibodies with FITC and Cy3 labeling were added and incubated for 1 h at room temperature protected from light. After 5 min of DAPI staining, cells or tissues were rinsed with PBS, blocked with an anti-fluorescent light quencher,0 and finally observed by fluorescence microscopy (Olympus, Japan).

### Western blotting

Cells and tissues were lysed with RIPA buffer containing protease inhibitors, total protein was extracted and protein concentrations were determined using the BCA kit. Protein samples were separated on 10% SDS polyacrylamide gels, while later transferred to PVDF membranes and closed with 5% skimmed milk powder before being incubated with anti-RIPK1 (1:1000), MLKL (1:1000, Abcam, ab243142), caspase-1 + p10 + p12 (1:1000, Abcam, ab179515), Gasdermin D (GSDMD) (1:1000, Abcam, ab219800), caspase-7 (1:100, Santa, sc-56063), caspase-3 (1:100, Santa, sc-56053), TAK1 (1:1000), GSK3β (1:1000, CST, #5676), ser9-GSK3β (1:1000, CST, #9322), TRAF6(1:100), HDAC3(1:1000), GAPDH(1:2000, Abcam, ab8245) and β-actin(1:2000, Abcam, ab8226) primary antibodies overnight at 4 °C. The next day the bands were washed by TBST and incubated with IRDye800CW secondary antibody for one hour protected from light using an Odyssey infrared imaging system (Li-COR Biosciences). After scanning, the membranes were washed for 5 min at room temperature with eluent and then closed for one hour with 5% skimmed milk powder. This was followed by incubation with GAPDH or β-actin in a shaker at 4 °C. The secondary antibody was incubated in the same way the next day and then scanned.

### Immunoprecipitation (IP) assay

Cells were lysed on ice with RIPA lysate containing protease inhibitors for 30 min, the supernatant was collected by centrifugation and the protein concentration was determined, a portion of the protein was retained and added to the loading buffer and boiled for 10 min. The remaining protein was incubated overnight at 4 °C with an anti-TRAF6 antibody (1:10) and normal immunoglobulin (IgG) was used as a negative IP control. The next day the beads were incubated with magnetic protein A/G beads at 4 °C for 4 h, washed with lysate, added to 1 × loading buffer and boiled at 100 °C for 15 min, together with the retained protein for subsequent protein blotting experiments.

#### Statistical analysis

Data analysis and drawing are carried out using GraphPadPrism 8.0 software. All data are reported as mean ± standard deviation (SD). Comparisons between groups were made using a one-way analysis of variance (ANOVA) or t-test. Differences were considered statistically significant when* P* < 0.05.

## Results

### GSK3β inhibitor TDZD-8 alleviates liver tissue structure and liver function damage in ALF mice

**Fig. 1:**
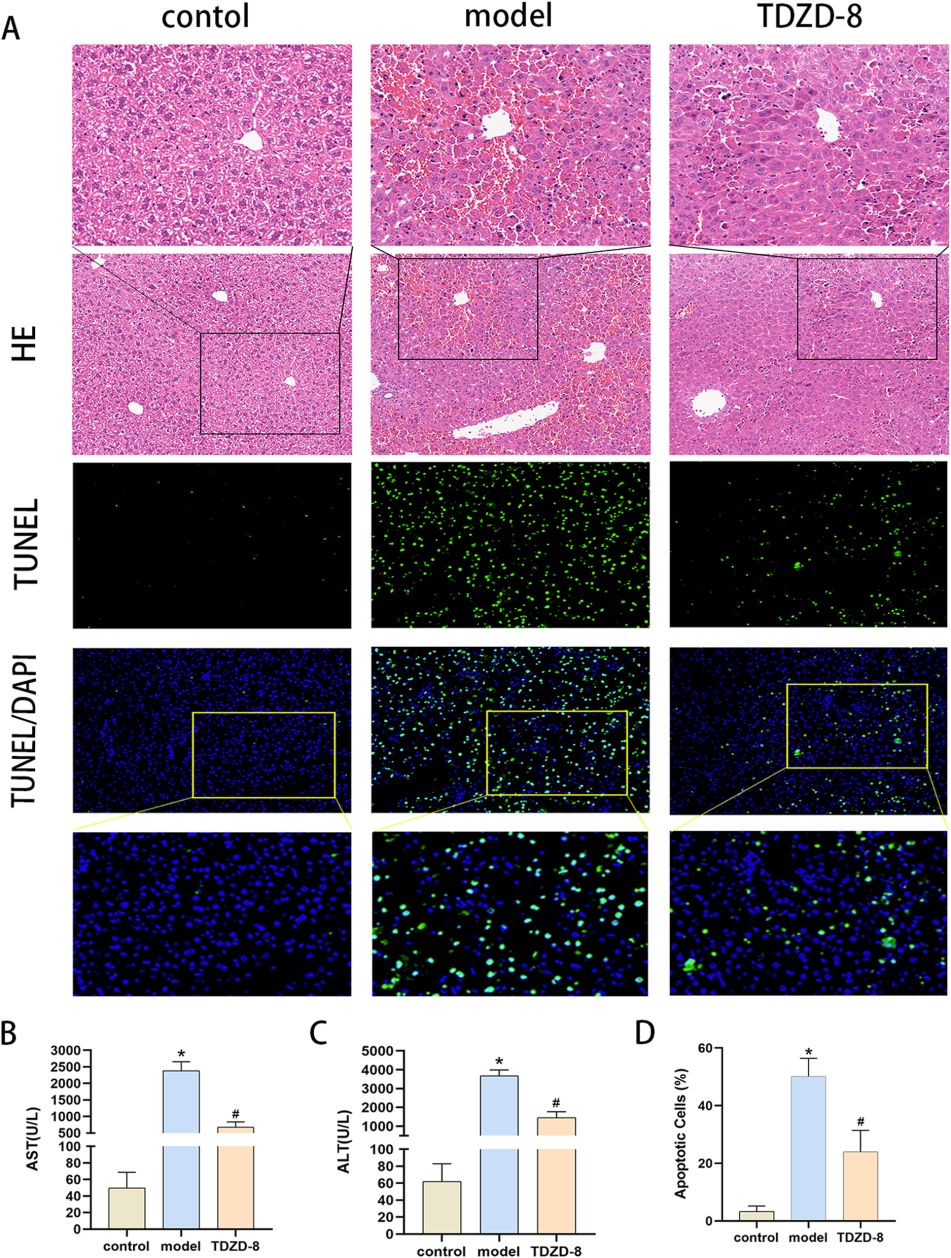
Effects of TDZD-8 on liver tissue injury, liver function and hepatocyte apoptosis in mice with acute liver failure. **A** Representative HE staining and TUNEL staining of liver tissue sections of each group of mice (magnification × 200). **B** Comparison of serum AST levels of mice in each group. **C** Changes in serum ALT levels of mice in each group. **D** The number of TUNEL-positive cells in the liver tissues of each group. The number of mice in each group was *n* = 6. ^*^P < 0.05 compared with control group, ^*#*^*P* < 0.05 compared with model group

In this experiment, an ALF model was constructed to verify the protective effect of TDZD-8 on ALF in mice. Compared with the control group, the model group showed obvious damage to the liver structure, disorganization of liver lobules and inflammatory cell infiltration, with a large number of hepatocyte necrosis and bruising; after treatment with TDZD-8, the liver structure was significantly improved, and hepatocyte necrosis and inflammatory cell infiltration were reduced (Fig. 1 A). At the same time, liver function was significantly impaired in the model group compared to the control mice, with significantly higher AST and ALT levels, and TDZD-8 treatment significantly reduced this abnormal increase (Fig. 1 B and C).

### TDZD-8 alleviates multiple programmed cell death levels in liver tissue of ALF mice

**Fig. 2:**
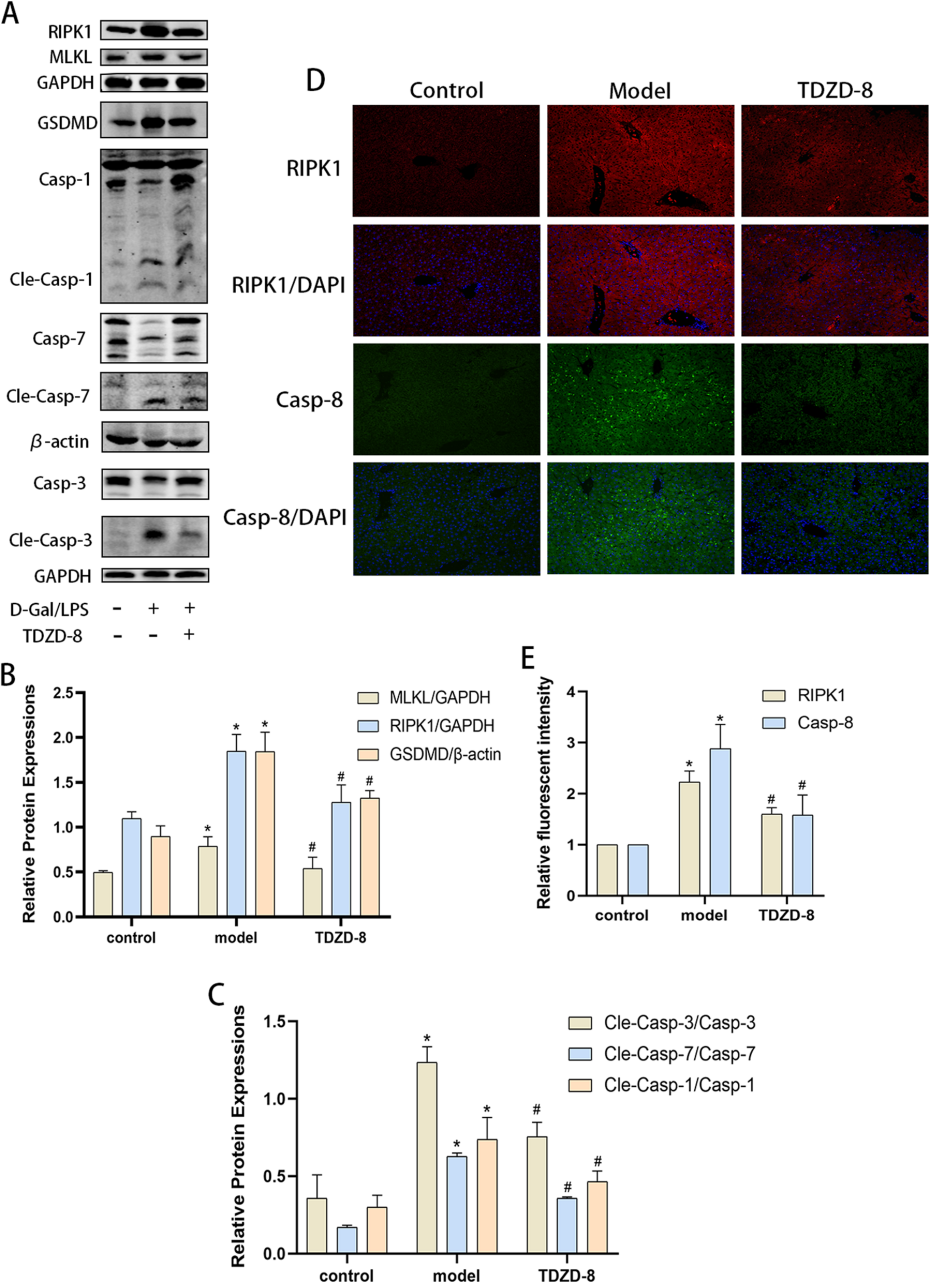
TDZD-8 treatment was able to reduce the levels of apoptosis, pyroptosis and necroptosis in liver tissues of mice with ALF. **A**-**C** Changes in MLKL, RIPK1, GSDMD, cleaved caspase-7, cleaved caspase-3 and cleaved caspase-1 protein levels in liver tissues. **D** and (**E**) Immunofluorescence detection of RIPK1 and caspase-8 localized expression and quantitative analysis (magnification × 200). ^*^P < 0.05 compare with control group, ^*#*^*P* < 0.05 compare with model group

Massive hepatocyte death is one of the main features of ALF. The level of apoptosis in liver tissue was shown using TUNEL staining (Fig. 1A and D). The number of apoptotic cells in liver tissue was significantly higher compared to the control group, while the number of positive apoptotic cells was significantly reduced after TDZD-8 treatment. Meanwhile, the expression levels of apoptosis, pyroptosis and necroptosis-related marker proteins were detected (Fig. 2A-C), and the levels of apoptosis-related cleaved caspase-3 and caspase-7, pro-pyroptosis protein cleaved caspase-1 and GSDMD and necroptosis-related protein RIPK1 and MLKL were significantly higher in the model group compared to the control group. However, the protein levels decreased after treatment with TDZD-8. In addition, the results of immunofluorescence detection of RIPK1 and caspase-8 expression in liver tissues were consistent with the protein blotting results (Fig. 2 D and E). These results indicated that TDZD-8 was able to alleviate the levels of apoptosis, pyroptosis and necroptosis in ALF mice.

### TDZD-8 alleviates the level of death of acutely injured hepatocytes in vitro

**Fig. 3:**
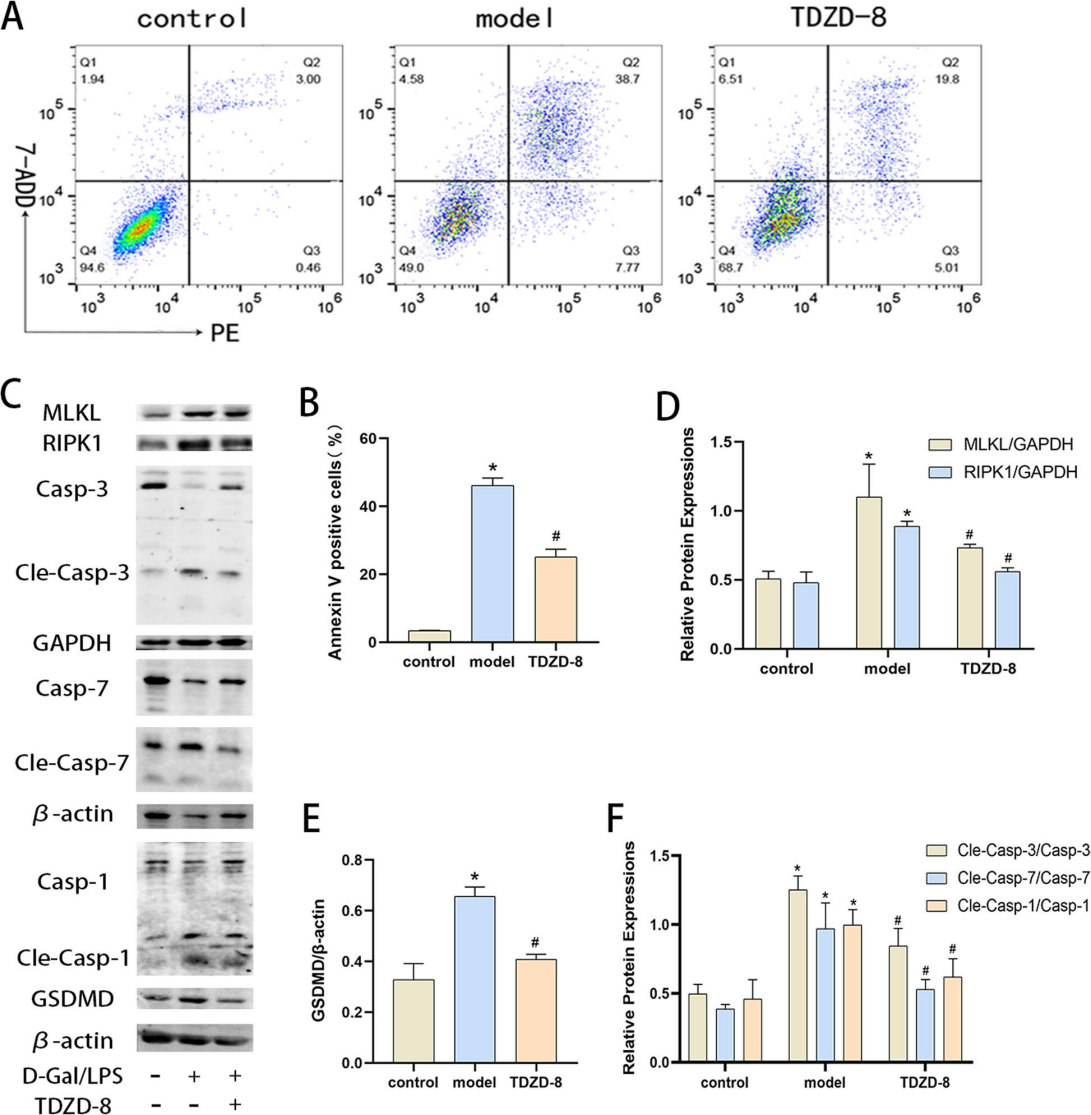
TDZD-8 alleviates the level of death in D-Gal/LPS stimulated cells. **A** and (**B**) the Percentage of apoptotic cells detected by flow cytometry. **C**-**F** Expression of MLKL, RIPK1, GSDMD, cleaved caspase-7, cleaved caspase-3 and cleaved caspase-1 protein in each group of cells and their quantitative analysis. ^*^P < 0.05 compare with control group, ^*#*^*P* < 0.05 compare with model group

Cells were stimulated with D-Gal/LPS to investigate the protective effect of TDZD-8 on acute hepatocyte injury in vitro. Flow cytometry showed that the apoptosis rate of the model group was significantly higher than that of the control group, and the apoptosis rate of the TDZD-8 group was significantly improved compared with that of the model group (Fig. 3 A and B). Protein blotting assayed the levels of apoptosis, pyroptosis and necroptosis-related proteins, and was consistent with the in vivo experiments. The levels of these proteins were significantly elevated in the model group compared to the control group, and the treatment with TDZD-8 significantly alleviated this abnormal alteration (Fig. 3 C-F). These results indicated that TDZD-8 was able to improve apoptosis, pyroptosis and necroptosis in acute hepatocyte injury in vitro.

### TDZD-8 inhibited GSK3β activity and altered TRAF6, HDAC3 and TAK1 levels in vivo and in vitro

**Fig. 4:**
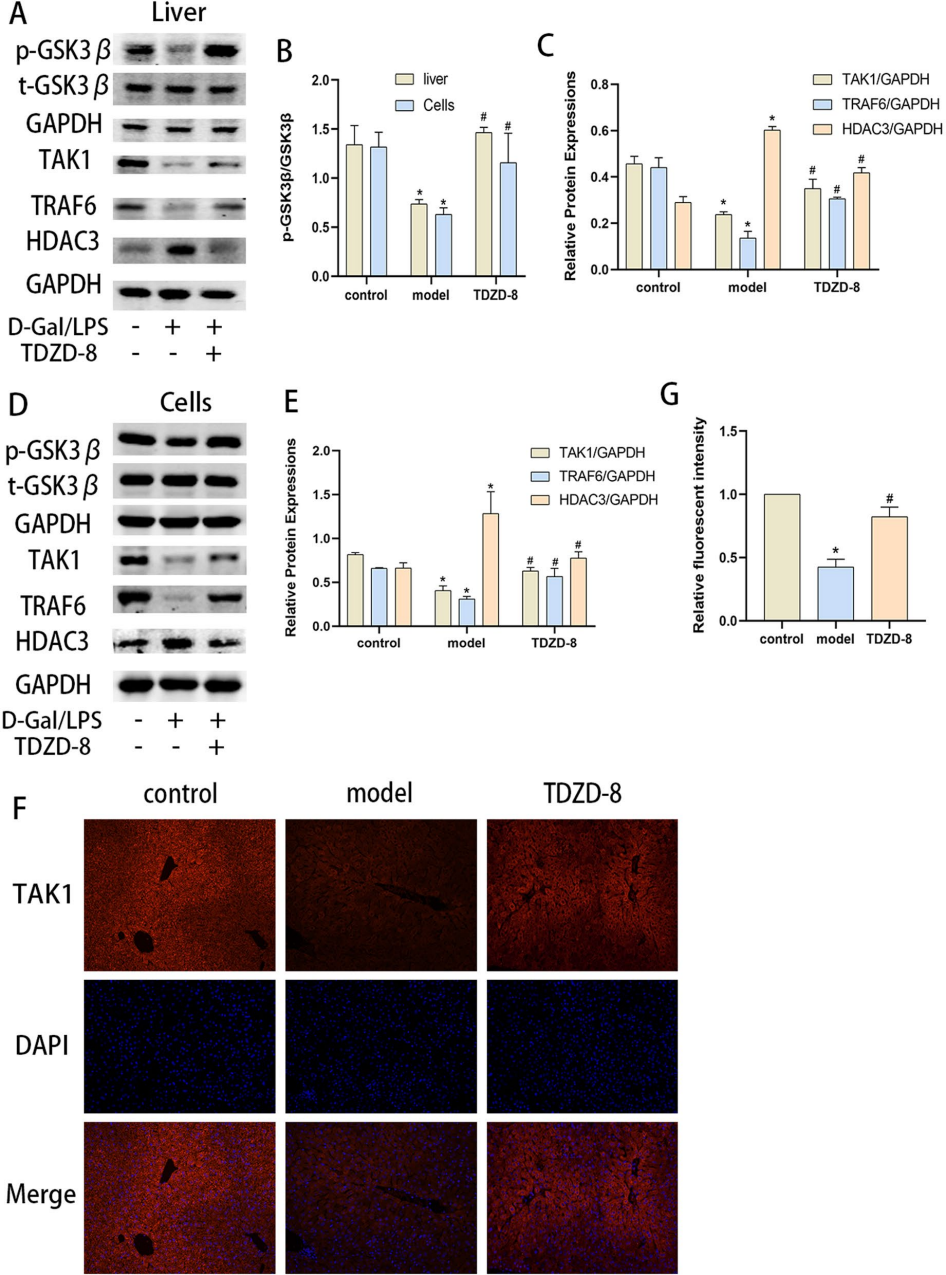
In vivo and in vitro, TDZD-8 treatment was able to regulate TRAF6, TAK1 and HDAC3 expression levels. **A**-**C** Expression of p-GSK3β, t-GSK3β, TAK1, TRAF6 and HDAC3 in liver tissues of each group of mice and their quantitative analysis. **B** and (**D**, **E**) Expression of p-GSK3β, t-GSK3β, TAK1, TRAF6 and HDAC3 in cells of each group and their quantitative analysis. **F** and (**G**) Immunofluorescence detection of localized expression of TAK1 in liver tissue and its mean fluorescence intensity statistics (magnification × 200). ^*^P < 0.05 compare with control group, ^*#*^*P* < 0.05 compare with model group

It has been shown that TAK1 plays a key role in the programmed mode of death in PANoptosis [14], and TRAF6 is one of the major activating molecules of TAK1 [18]. We then examined the protein levels of TAK1, TRAF6, and HDAC3 using protein blotting assays and found that the p-GSK3β ratio decreased in the model group, and the protein levels of TAK1 and TRAF6 decreased, but HDAC3 levels increased, in vivo and in vitro. In contrast, TDZD-8 was able to up-regulate TAK1 and TRAF6 levels and decrease HDAC3 expression compared to the model group while increasing the p-GSK3β ratio (Fig. 4 A-E). The results of immunofluorescence detection of TAK1 localization and expression in liver tissues were consistent with the protein blotting results (Fig. 4 F and G). For this reason, TDZD-8 may exert a protective effect by altering the levels of TAK1, HDAC3 and TAK1.

### Inhibition of TRAF6 partially reverses the protective effect of TDZD-8 in vitro

**Fig. 5:**
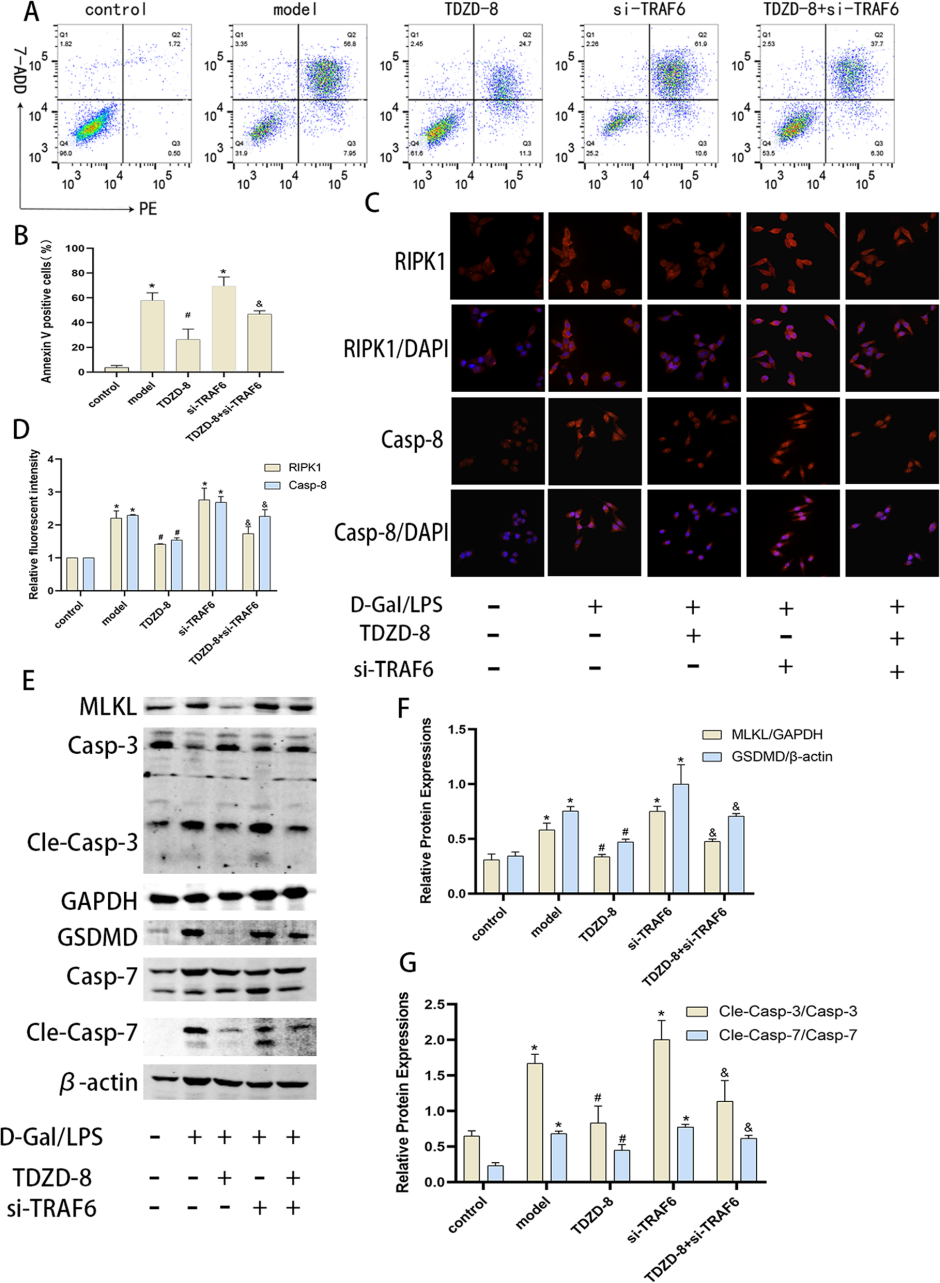
Inhibition of TRAF6 was able to partially reverse the hepatocyte-protective effect of TDZD-8 in vitro. **A** and (**B**) Flow cytometry detection of apoptosis levels in each group and its statistical analysis. **C** and (**D**) Immunofluorescence detection of RIPK1 and caspase-8 localization and expression levels in vitro and their quantitative analysis (magnification × 400). **E**–**G** Protein blotting and immunofluorescence to detect MLKL, GSDMD, cleaved caspase-7, cleaved caspase-3 protein levels in each group of cells and their quantitative analysis. ^*^P < 0.05 compare with control group, ^*#*^*P* < 0.05 compare with model group, ^*&*^*P* < 0.05 compare with TDZD-8 group

To further investigate the role of TRAF6 in the cytoprotective process of TDZD-8, we used si-RNA to inhibit TRAF6 expression. As expected, the therapeutic effect of TDZD-8 was reduced after the inhibition of TRAF6. Flow cytometry showed that after inhibition of TRAF6 expression, the apoptosis rate was able to increase compared to the model group, and after combined treatment with TDZD-8 the apoptosis rate increased significantly compared to TDZD-8 alone (Fig. 5 A and B). In addition, protein blotting and immunofluorescence detection of apoptosis, pyroptosis and necroptosis-related protein expression revealed that inhibition of TRAF6 did not down-regulate cell death protein expression, but even up-regulated cell death protein expression after TDZD-8 treatment, partially reversing the protective effect of TDZD-8 (Fig. 5 C-G). The above results suggest that inhibition of GSK3β can function through the TRAF6 molecule.

### TRAF6 interacts with HDAC3 to reduce HDAC3 molecular levels

**Fig. 6:**
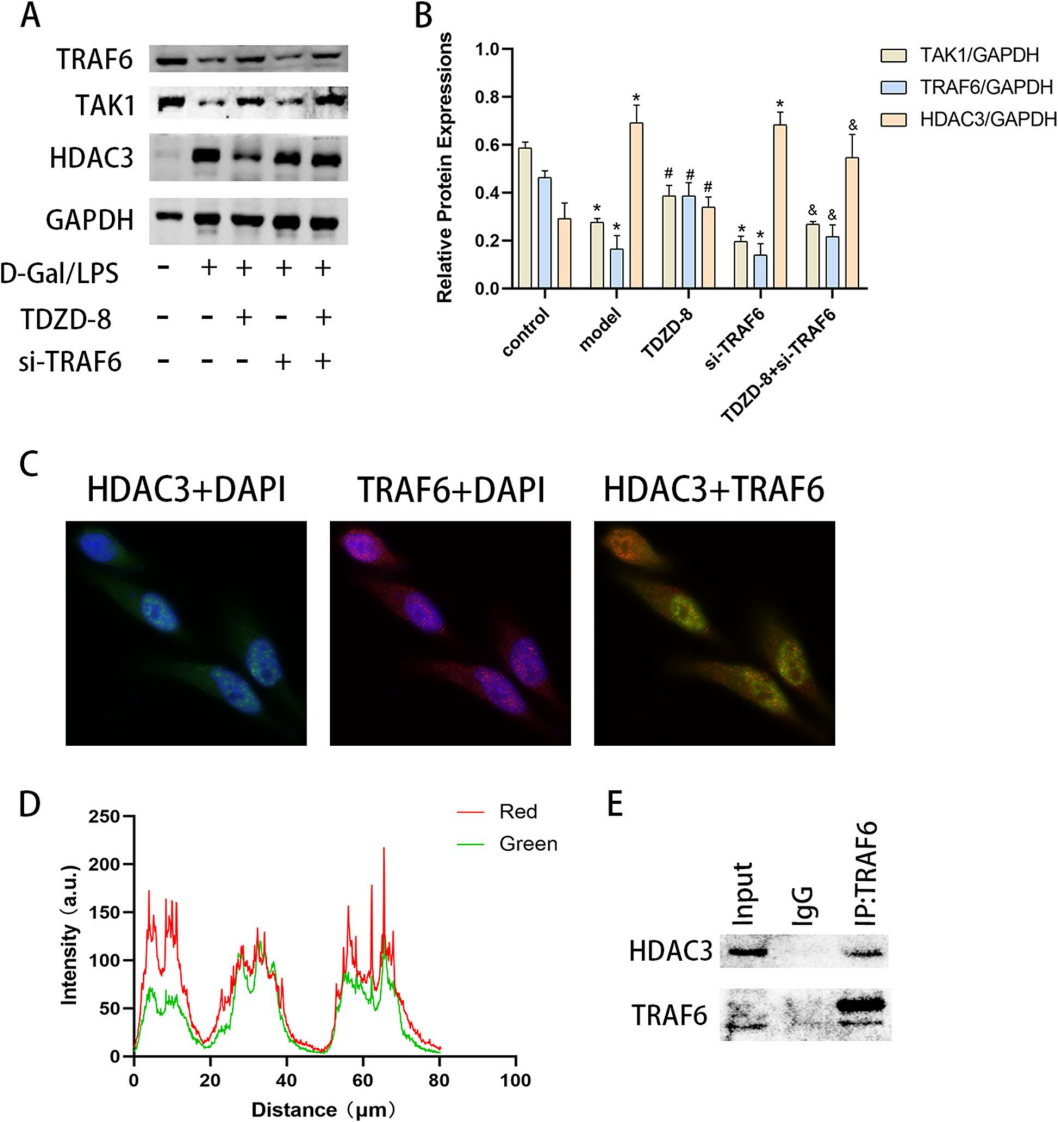
TRAF6 can regulate TAK1 while being able to interact with HDAC3 to alter HDAC3 levels. **A** and (**B**) Protein blotting to detect TRAF6, TAK1 and HDAC3 protein levels in vitro and their quantitative analysis. **C** and (**D**) Immunofluorescence detection of TRAF6 and HDAC3 co-localization in vitro and its statistical analysis (magnification × 1000). **E** Immunoprecipitation assay to detect the interaction of HDAC3 with TRAF6. ^*^P < 0.05 compare with control group, ^*#*^*P* < 0.05 compare with model group, ^*&*^*P* < 0.05 compare with TDZD-8 group

Recent studies have revealed that TRAF6 is able to interact with HDAC3 molecules, ubiquitinate to reduce HDAC3 levels, upregulate proto-oncogenes and promote hepatocellular carcinogenesis [19]. In the present experiments, inhibition of GSK3β activity increased TAK1 levels and decreased HDAC3 levels, but inhibition of TRAF6 expression levels decreased the effect of TDZD-8, and in contrast to TDZD-8 alone, inhibition of TRAF6 decreased TAK1 levels and increased HDAC3 levels (Fig. 6 A and B). Using fluorescence co-localization analysis, it was found that HDAC3 and TRAF6 shared a common expression site in cells, and interestingly, HDAC3 expression was relatively low at sites of high TRAF6 expression (Fig. 6 C and D). Furthermore, the interaction between the two molecules in cells was detected using immunoprecipitation methods and was found to be present in normal hepatocytes (Fig. 6 E). In vitro experiments suggest that TRAF6 has some interaction with HDAC3 in hepatocytes and can reduce HDAC3 expression levels.

### HDAC3 inhibition exerts hepatoprotective effects via TAK1

**Fig. 7:**
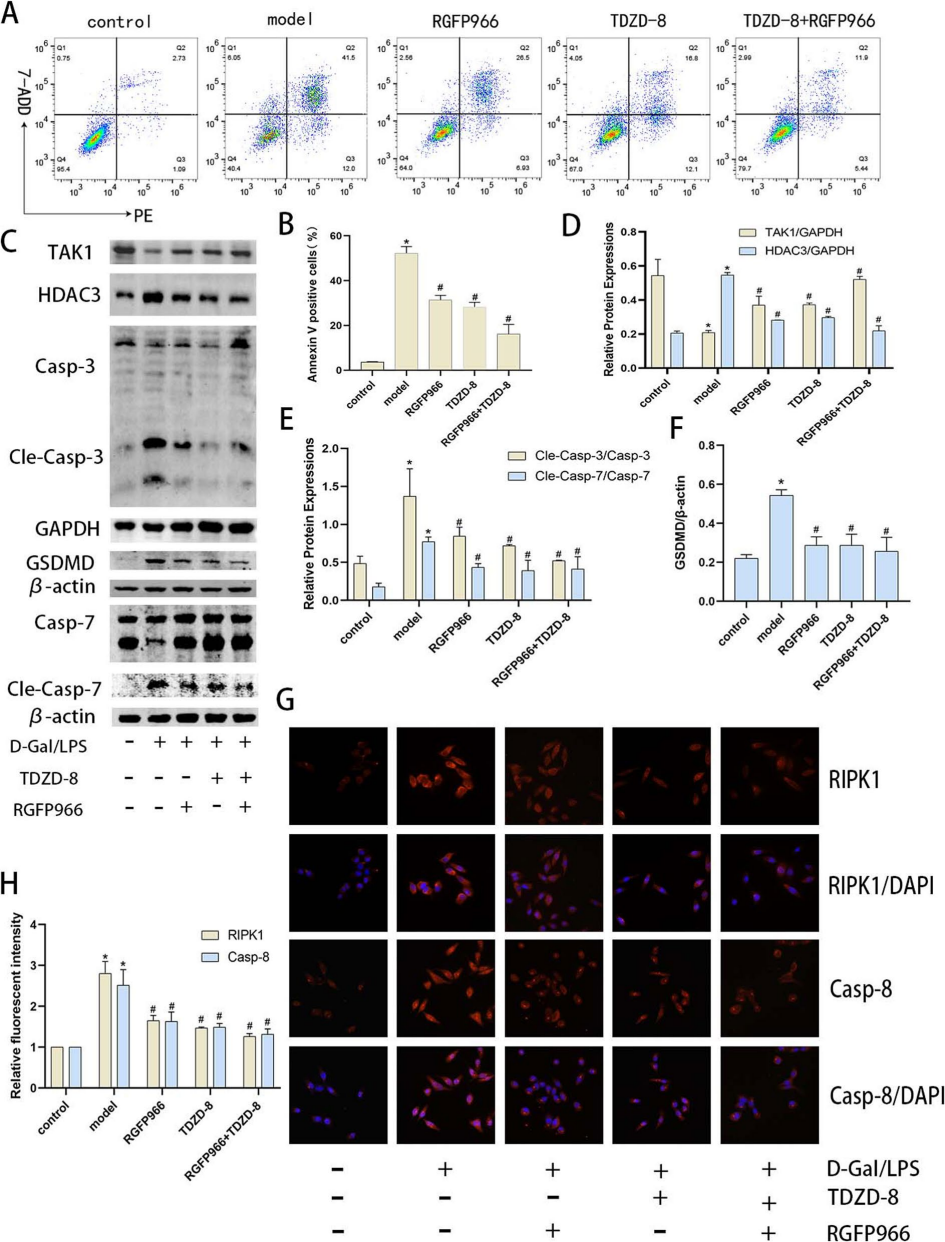
Inhibition of HDAC3 levels modulates TAK1 levels and attenuates the level of death in acute hepatocyte injury. **A** and (**B**) the Percentage of apoptotic cells in each group by flow cytometry. **C**-**F** Protein blotting of TAK1, HDAC3, GSDMD, cleaved caspase-7 and cleaved caspase-3 protein expression in vitro and quantitative analysis. **G** and (**H**) Immunofluorescence detection of RIPK1 and caspase-8 localization and expression in vitro (magnification × 400). ^*^P < 0.05 compare with control group, ^*#*^*P* < 0.05 compare with model group

**Fig. 8:**
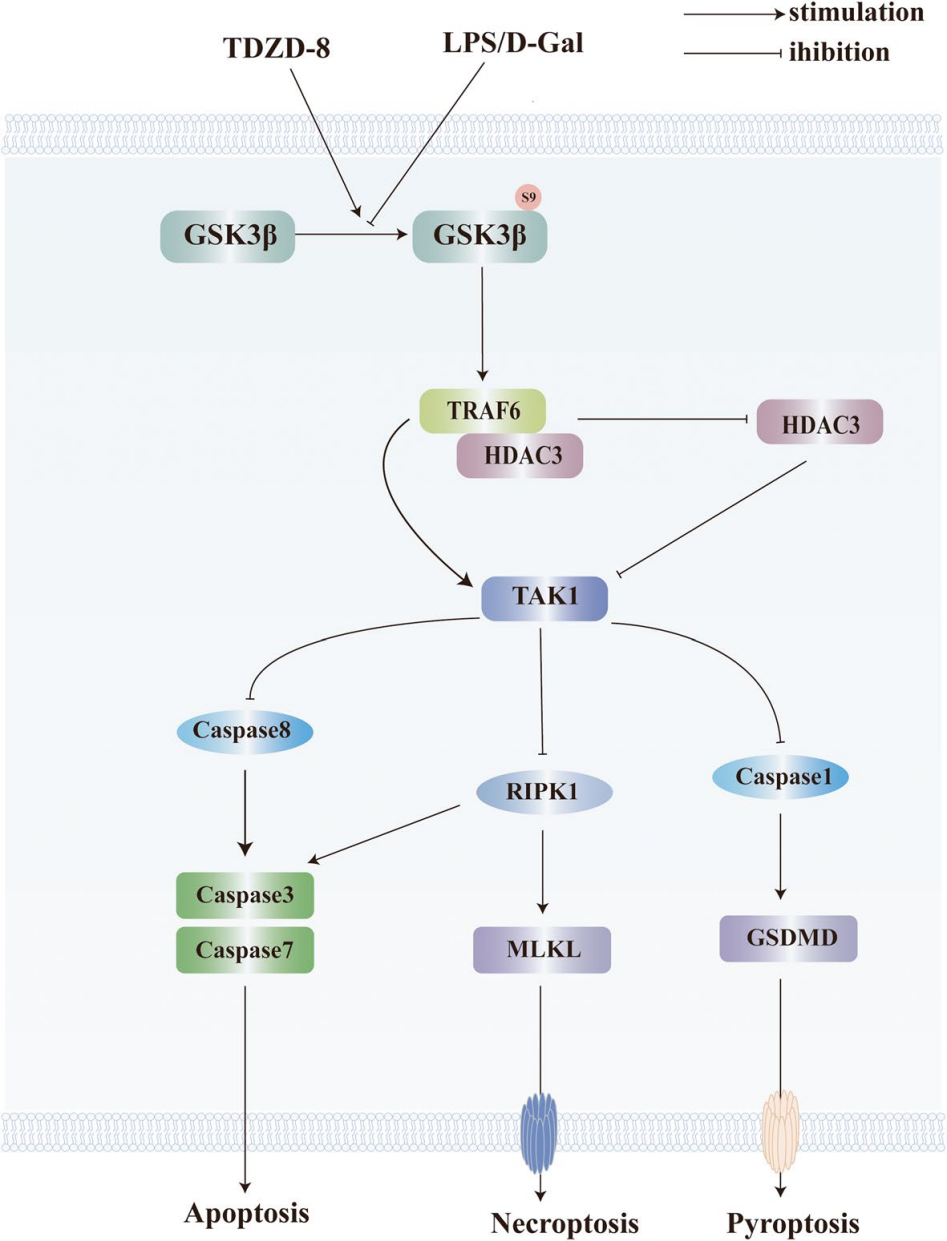
The schematic shows that the GSK3β inhibitor TDZD-8 ameliorates cell death via the TAK1 pathway. the combination of LPS/D-Gal activates GSK3β and reduces TRAF6 levels, thereby increasing HDAC3 levels and reducing TAK1, promoting hepatocyte apoptosis, pyroptosis and necroptosis

An increasing number of experiments have demonstrated that inhibition of HDAC can exert hepatoprotective effects by inhibiting apoptosis, pyroptosis and oxidative stress. It was found by flow cytometry that inhibition of HDAC3 was able to significantly reduce the rate of apoptosis in hepatocytes (Fig. 7 A and B). Protein blotting and immunofluorescence detection of pro-cell death-related proteins revealed that inhibition of HDAC3 increased TAK1 levels but significantly decreased pro-cell death-related protein expression and reduced hepatocyte death (Fig. 7 C-H). In conclusion, we found that inhibition of GSK3β activity could improve apoptosis, pyroptosis and necroptosis through TRAF6/HDAC3/TAK1 pathway and exert hepatocyte protective effects Fig. 8.

## Discussion

This study focuses on the hepatoprotective effects and potential mechanisms of the GSK3β inhibitor TDZD-8 in a mouse model of acute liver failure, the LPS/D-Gal mouse model of liver failure, which is used not only to study acute liver failure but also as a study of fulminant liver failure, acute liver injury and other liver diseases [20, 21]. In previous studies, liver injury in this model was caused by the involvement of various complex mechanisms such as excessive inflammatory response, oxidative stress, apoptosis, necrosis, autophagy, pyroptosis, ferroptosis, etc. [22–25]. The various mechanisms interacted and promoted each other, for example, excessive inflammatory response deteriorated liver function and promoted hepatocyte necrosis and apoptosis; in turn, the large number of necrotic liver cells further contributed to the inflammatory response of the liver [3]. However, most studies have examined only one or two mechanisms, reporting on various hepatoprotective agents and analyzing their protective mechanisms. It is because the factors and mechanisms of liver injury are intricate and interact with each other that it is more helpful to study substances that can control multiple mechanisms simultaneously in acute liver failure. Various stimuli damage induces cells to form tumor necrosis factor receptor (TNFR) complex I containing RIPK1, which is capable of promoting apoptosis both through caspase-8 activation and through RIPK1 and receptor-interacting protein kinase 3 (RIPK3) activation promoting MLKL oligomerization and translocation to the cell membrane, thereby promoting necroptosis [26, 27]. Therefore, RIPK1 is at the pivotal position between apoptosis and necroptosis and can promote different types of apoptosis through different pathways [28]. Regardless of upstream signaling, phosphorylation of MLKL is a central event in necroptosis [29]. More and more experiments have revealed that the gasdermin protein family plays an important role in scorch death occurrence. In particular, GSDMD is considered a key executor of pyroptosis and an essential molecule in pyroptosis [30]. In this study, a mouse model of acute liver failure was constructed by intraperitoneal injection of LPS and D-Gal. As expected, liver tissue sections from ALF mice showed significant necrosis and inflammatory cell infiltration, and apoptosis levels were significantly increased by TUNEL staining. At the same time, the levels of apoptosis, necrosis and pyroptosis-related proteins in mouse liver tissues were also significantly elevated. However, TDZD-8 treatment decreased liver injury and liver function in mice, while downregulating the levels of apoptosis, necrosis and pyroptosis-related markers. Similarly, we obtained consistent results in an in vitro model. In our previous study, TDZD-8 was able to improve the inflammatory response in mice with acute liver failure and exerted hepatoprotective effects. In addition, inhibition of GSK3β has been shown to alleviate liver inflammation, reduce hepatocyte apoptosis and activate hepatocyte autophagy, improving liver function and exerting hepatoprotective effects in previous studies. Moreover, it has been shown that GSK3β interacts with RIPK1 molecules to promote RIPK1 molecular activity and promote post-stroke scar formation, leading to poor prognosis [11].

In several studies, TAK1 is one of the major regulatory molecules of pyroptosis, apoptosis and necroptosis. In the absence or inhibition of TAK1 activity, cells are prone to initiate programmed cell death in response to external stimuli [31]. TAK1 is a key molecule in the regulation of inflammation and cell death and plays a critical role in regulating cell survival, differentiation and apoptosis. TAK1 activates NF-κB, promotes transcription of downstream anti-apoptotic proteins and promotes cell growth and proliferation [32]. At the same time, inhibition or deletion of TAK1 can initiate RIPK1-mediated apoptosis or RIPK1-RIPK3-mediated necroptosis, promoting programmed cell death in a variety of cells. Not only that, in recent years, it has been shown that TAK1 acts as a direct upstream activator of AMPK, inducing protective autophagy and regulating cell death [33]. Moreover, hepatocyte-specific deficiency of TAK1 can activate RIPK1-dependent inflammatory responses in hepatocytes, promoting hepatocellular fibrosis and hepatocellular carcinogenesis and facilitating disease progression [34]. Given this, we speculated whether the marked apoptosis, scorch death and necroptosis in the ALF model were associated with decreased TAK1 levels. To this end, we examined changes in TAK1 content in mouse liver tissues and cells, and we found that TAK1 content was significantly downregulated in the induced ALF model, but increased after TDZD-8 treatment as GSK3β activity was inhibited. Therefore, we suggest that inhibition of GSK3β can improve ALF through TAK1.

Subsequently, we further investigated the specific mechanisms by which inhibition of GSK3β activity ameliorates multiple programmed cell deaths via TAK1 in ALF models. Many studies have shown that TRAF6 is a typical activator of TAK1 with an N-terminal cyclic domain that functions as an E3 ubiquitin ligase and that TRAF6 generates a lysine-63 (K63)-Ub oligomer that activates TAK1 in vitro [18]. In addition to the typical NF-κB pathway, TRAF6 and TAK1 play key roles in the development and homeostasis of several tissues by regulating cell survival, proliferation and differentiation. In one study, using a cinchonine drug capable of targeting the TRAF6 molecule, ubiquitination reduced AKT while inhibiting TAK1 activity and promoting apoptosis in cancer cells [35]. TRAF6 is a bridging protein that mediates multiple signaling pathways and is overexpressed in many tumor cells, activating the AKT signaling pathway and promoting cell survival and migration [36]. TRAF6 interacts with Deltex to induce cysteine-mediated apoptosis and promote cell death [37]. In addition, it has also been shown that reduced TRAF6 expression levels correlate with increased GSK3β protein levels and activity. TRAF6 is then phosphorylated by GSK3β at Thr266, which promotes K48-linked polyubiquitination and degradation of TRAF6, thereby reducing TRAF6 molecular levels [38]. To further verify the role of TRAF6, we reduced TRAF6 expression in cells and examined the levels of TAK1 and various programmed cell death-related proteins and found that reducing TRAF6 did not reduce hepatocyte injury, but even reduced TAK1 levels, attenuated the protective effect of TDZD-8 on hepatocytes and increased cell death protein levels after TDZD-8 treatment. Therefore, we suggest that inhibition of GSK3β may act by altering TRAF6 molecular content and activity.

The E3 ubiquitin ligase activity of TRAF6 has also been shown to ubiquitously downregulate HDAC3 levels and upregulate c-Myc gene expression, promoting hepatocellular carcinogenesis and poor prognosis [19]. HDAC3 is a class I histone deacetylase that regulates chromatin structure and gene expression, mainly found in the nucleus. Histone deacetylase inhibitors play a key role in tumor therapy [39]. HDAC has been shown to regulate the GSK3β pathway involved in disease progression [40]. Recent studies have shown that dual HDAC/GSK3β inhibition therapy promotes neuronal survival and controls tumor growth [41, 42]. Given this, we speculated whether changes in TRAF6 levels in this model could alter HDAC3 and play a role. In this experiment, we first examined the changes in HDAC3 levels in the model liver and cells, and found that HDAC3 levels increased significantly in the model, and TDZD-8 treatment significantly reduced its levels, suggesting that altering GSK3β activity could regulate HDAC3 levels; then we used siRNA to alter TRAF6 expression levels, and found that inhibition of TRAF6 expression partially reversed the effect of TDZD-8 on HDAC3. The effect of TDZD-8 was partially reversed and HDAC3 levels were increased. We verified the co-localized expression of HDAC3 and TRAF6 in cells by fluorescence microscopy, while immunoprecipitation experiments demonstrated that TRAF6 molecules can interact with HDAC3 to alter HDAC3 levels and thus increase TAK1 levels to improve ALF. To verify the role of HDAC3, we used HDAC3-specific inhibitors to reduce HDAC3 in cells We found that lowering HDAC3 simultaneously significantly increased TAK1 levels and improved cell injury and reduced death levels. Thus, inhibition of GSK3β activity could improve cell death levels in acute liver failure by modulating the activity levels of the TRAF6/HDAC3/TAK1 molecular pathway. However, the exact mechanism of how HDAC3 alters the level of TAK1 activity needs to be further investigated.

In summary, our experiments show that TDZD-8 can inhibit GSK3β activity and exert partial hepatoprotective effects, mainly by alleviating hepatocyte apoptosis and possibly acting through the TRAF6/HDAC3/TAK1 pathway. TDZD-8 pretreatment reduces liver damage and decreases the inflammatory response of the liver, thus making it a potential prophylactic agent for the treatment of acute liver failure. However, it is of concern that our current work is focused on the preventive effect of GSK3β inhibition on acute liver failure, although some studies have shown that TDZD-8 has some preventive and therapeutic value in ischemia–reperfusion injury [43]. Still, further studies are needed to explore the therapeutic effects of applying specific GSK3β inhibitors after acute liver failure, and the potential therapeutic window for the administration of inhibitors after injury.

### Supplementary Information

**Additional file 1.** 

## Data Availability

The data of the study are available on request from the author, DZ.
