# Correction: Inhibition of GSK3β activity alleviates acute liver failure via suppressing multiple programmed cell death

**DOI:** 10.1186/s12950-024-00390-1

**Published:** 2024-06-12

**Authors:** Danmei Zhang, Chunxia Shi, Qingqi Zhang, Yukun Wang, Jin Guo, Zuojiong Gong

**Affiliations:** Department of Infectious Diseases, Renmin Hospital of Wuhan University, No. 238 Jiefang Road, Wuhan, 430060 Hubei Province China

**Correction: J Inflam 20, 24 (2023)**

**https://doi.org/10.1186/s12950-023-00350-1**

After publication of this article, it was reported that in Figs. 3, 5 and 7, some errors occurred; the figures should have appeared as shown below.

The first is a set of cellular immunofluorescence images of CAS8 molecules in Fig. 5C. The first two merge images were inverted due to negligence. Secondly, regarding the statistical analysis of the apoptosis flow pattern in Figs. 3, 5 and 7, it was not the intention to analyze the early apoptotic cell population as an apoptosis indicator to support the conclusion. However, after carefully discussing and reviewing two instructions from BD and Abcam, and based on previous studies [1, 2], it would be more rigorous to change the vertical coordinate of the graph to Annexin V positive cells (%) instead of apoptosis rate in Figs. 3, 5 and 7, which would only illustrate the results of the experiments.

**Fig. 3:**
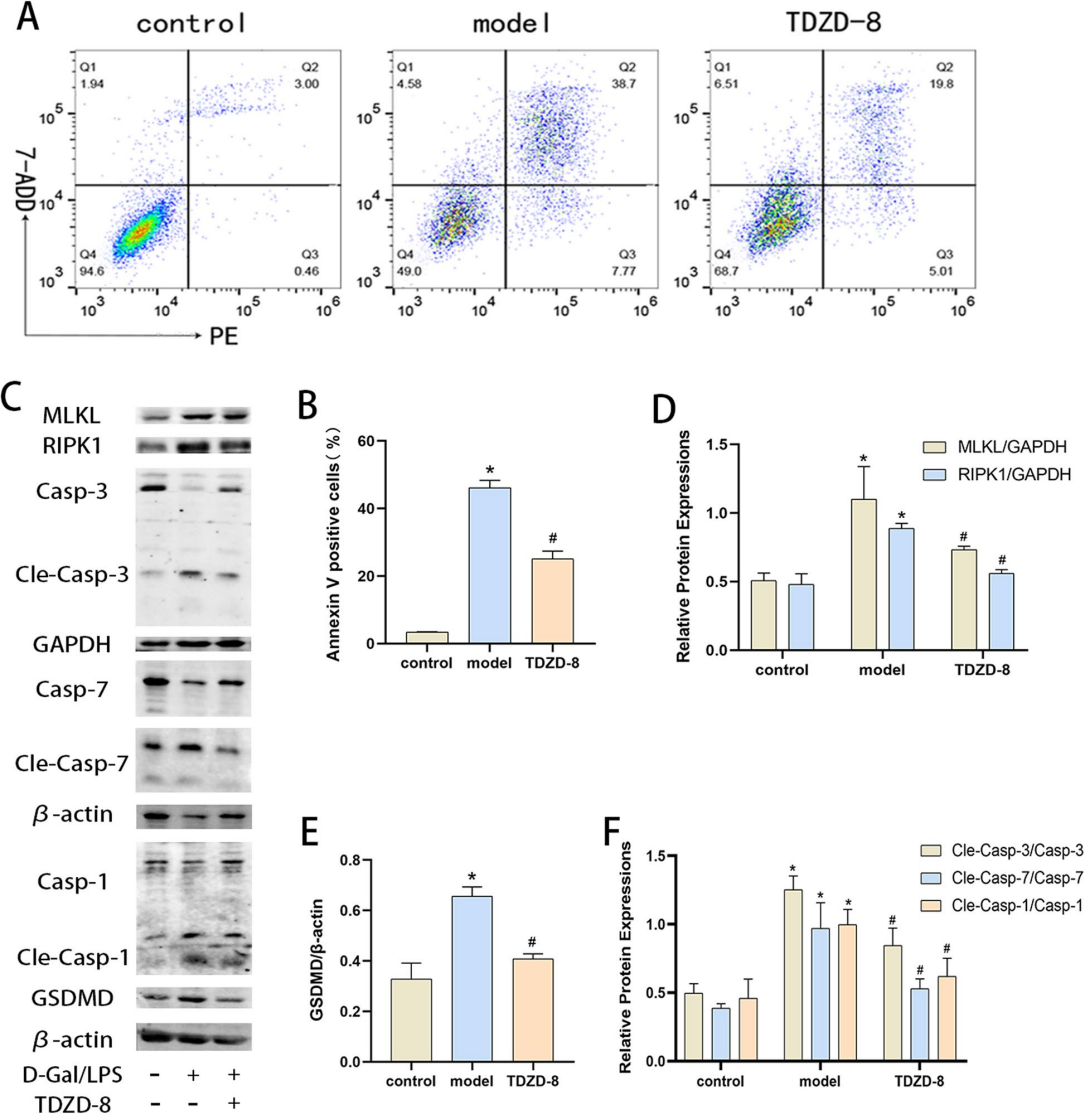
TDZD-8 alleviates the level of death in D-Gal/LPS stimulated cells. **A** and (**B**) the Percentage of apoptotic cells detected by flow cytometry. **C-F** Expression of MLKL, RIPK1, GSDMD, cleaved caspase-7, cleaved caspase-3 and cleaved caspase-1 protein in each group of cells and their quantitative analysis. *P < 0.05 compare with control group, *#P* < 0.05 compare with model group

**Fig. 5:**
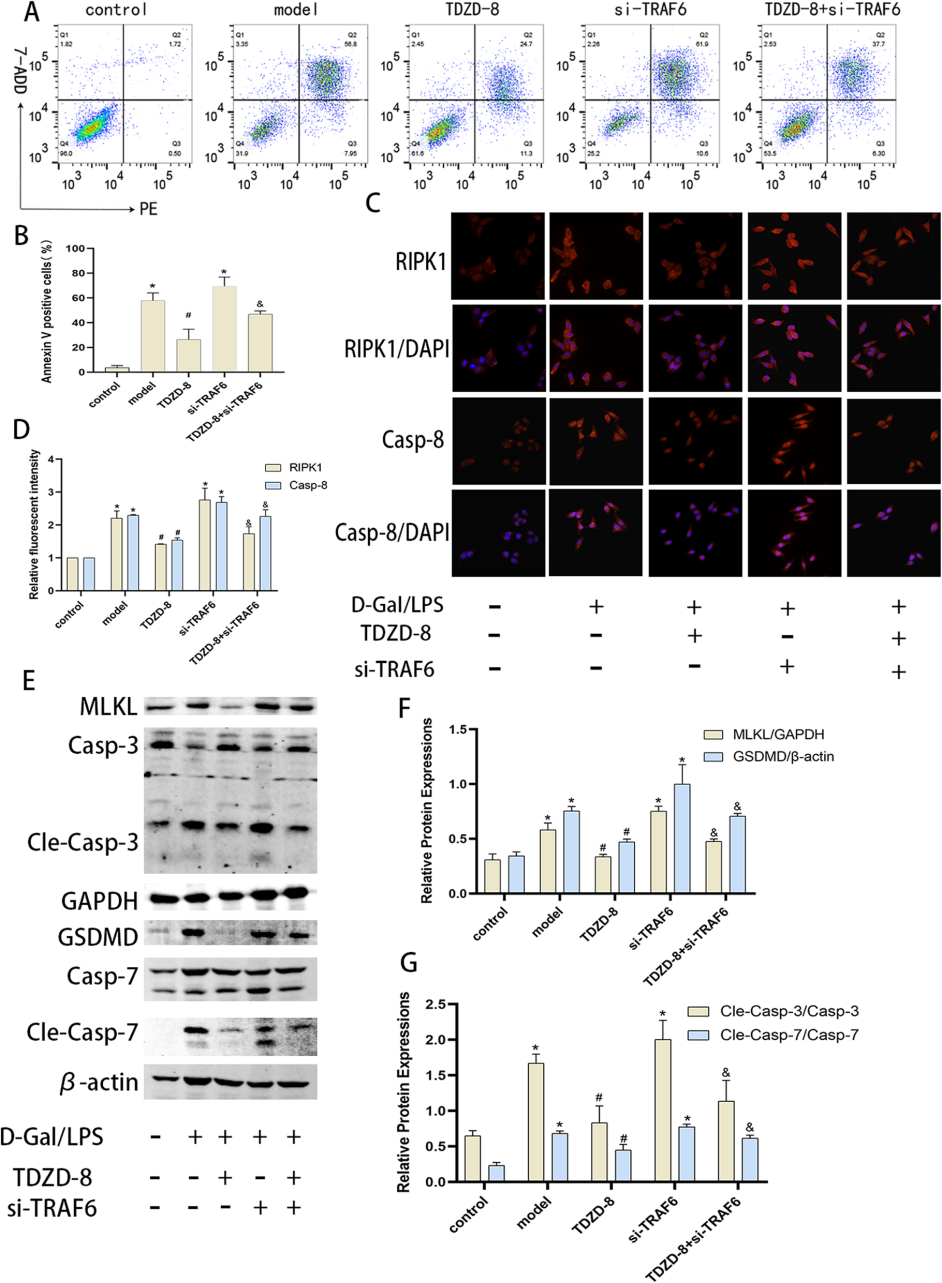
Inhibition of TRAF6 was able to partially reverse the hepatocyte-protective effect of TDZD-8 in vitro. **A** and (**B**) Flow cytometry detection of apoptosis levels in each group and its statistical analysis. **C** and (**D**) Immunofluorescence detection of RIPK1 and caspase-8 localization and expression levels in vitro and their quantitative analysis (magnification × 400). **E–G** Protein blotting and immunofluorescence to detect MLKL, GSDMD, cleaved caspase-7, cleaved caspase-3 protein levels in each group of cells and their quantitative analysis. *P < 0.05 compare with control group, *#P* < 0.05 compare with model group, *&P* < 0.05 compare with TDZD-8 group

**Fig. 7:**
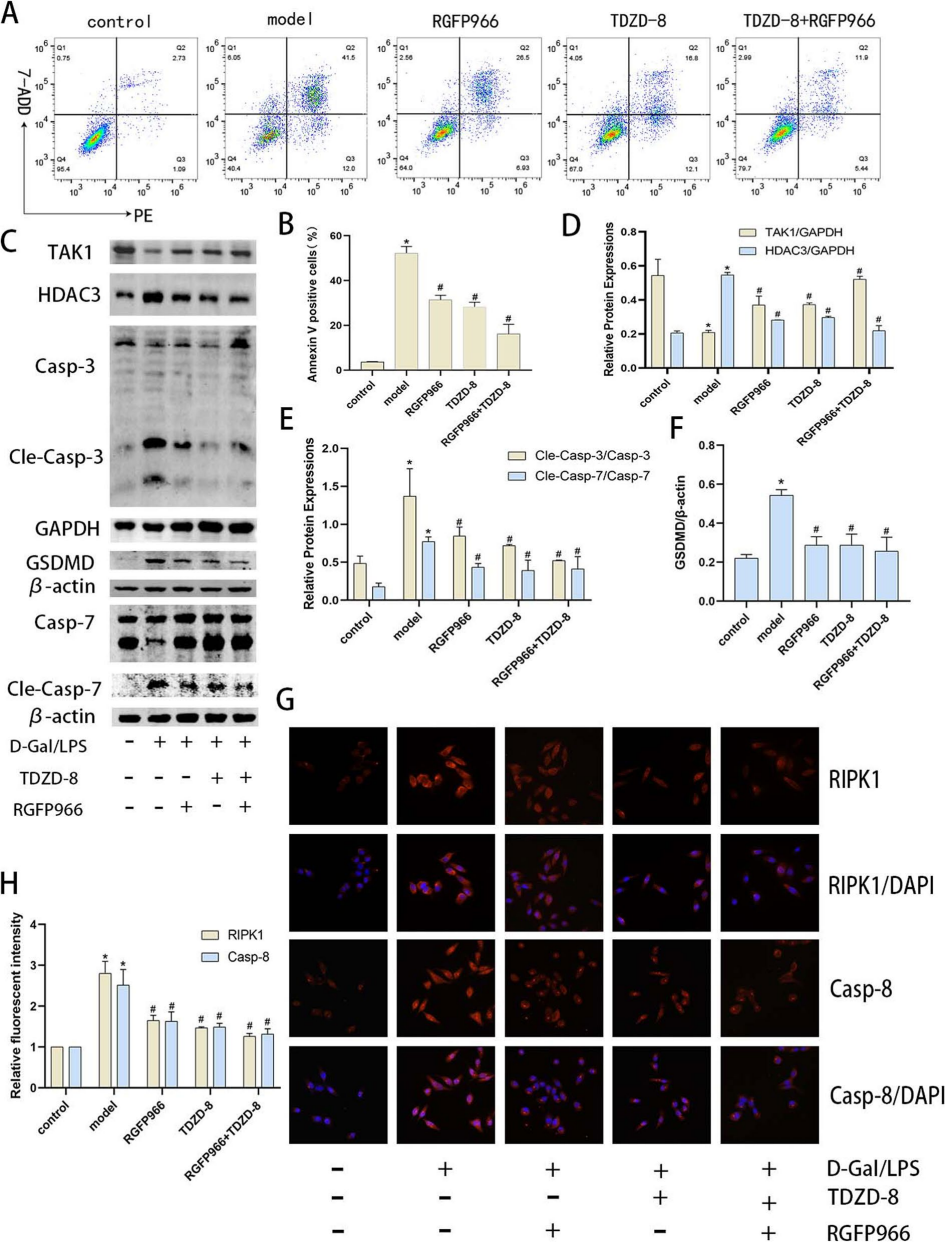
Inhibition of HDAC3 levels modulates TAK1 levels and attenuates the level of death in acute hepatocyte injury. **A** and (**B**) the Percentage of apoptotic cells in each group by flow cytometry. **C-F** Protein blotting of TAK1, HDAC3, GSDMD, cleaved caspase-7 and cleaved caspase-3 protein expression in vitro and quantitative analysis. **G** and (**H**) Immunofluorescence detection of RIPK1 and caspase-8 localization and expression in vitro (magnification × 400). *P < 0.05 compare with control group, *#P* < 0.05 compare with model group

The original article has been updated by the authors.
