# Supplementary material for: Inhibition of GSK3β activity alleviates acute liver failure via suppressing multiple programmed cell death
Source: J Inflamm (Lond). 2023 Jul 13;20:24. doi: 10.1186/s12950-023-00350-1 (PMC10347874; doi:10.1186/s12950-023-00350-1)

**Inhibition of GSK3β activity alleviates acute liver failure via suppressing multiple programmed cell death**

Danmei Zhang, Chunxia Shi, Qingqi Zhang, Yukun Wang, Jin Guo, Zuojiong Gong

Department of Infectious Diseases, Renmin Hospital of Wuhan University, 430060, Wuhan, China

# Corresponding author: Zuojiong Gong; Department of Infectious Diseases, Renmin Hospital of Wuhan University, 238 Jiefang Road, Wuhan, 430060, China. E-mail: [zjgong@163.com](mailto:zjgong@163.com)

**WB**

**Figure 2 A**

**Repeat 1**


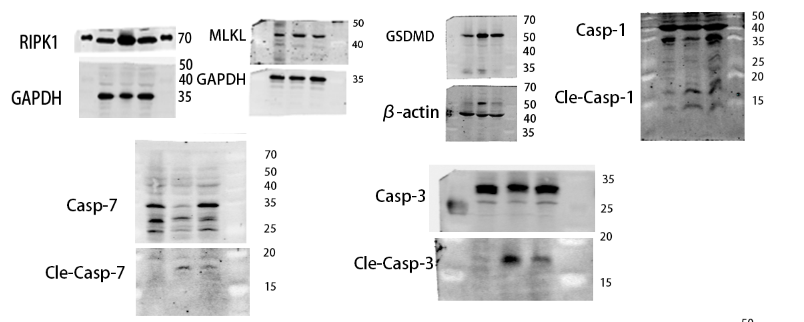


**Repeat 2**


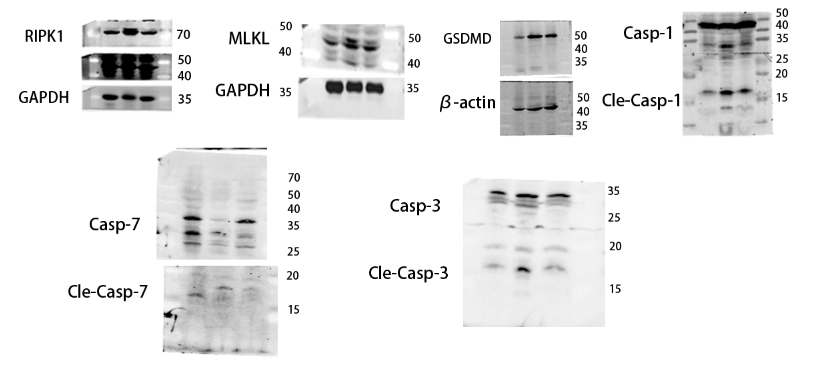


**Repeat 3**


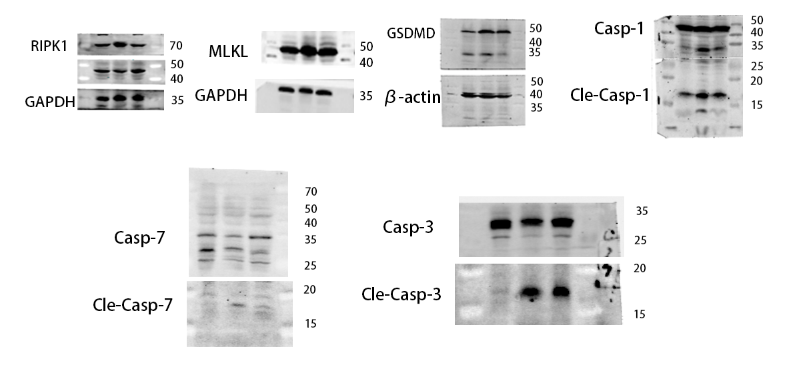


**Note: After scanning the GSDMD with the membrane sweeper, the membrane was washed with eluent, closed and incubated with β-actin. The target molecule was located on the same membrane as the β-actin.**

**Figure 3 C**

**Repeat 1**


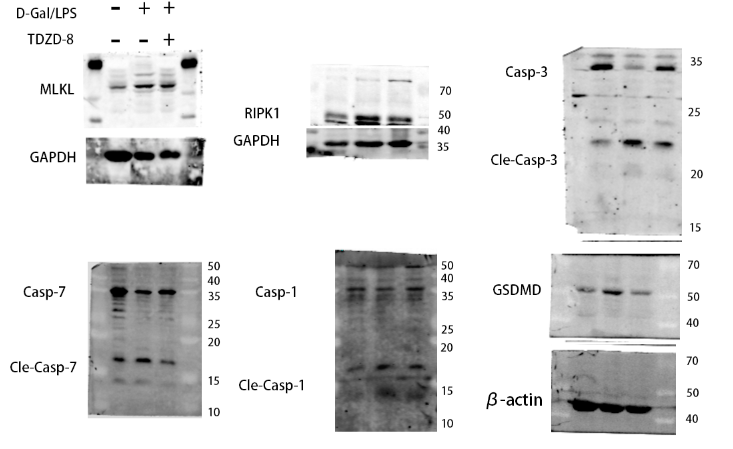


**Repeat 2**


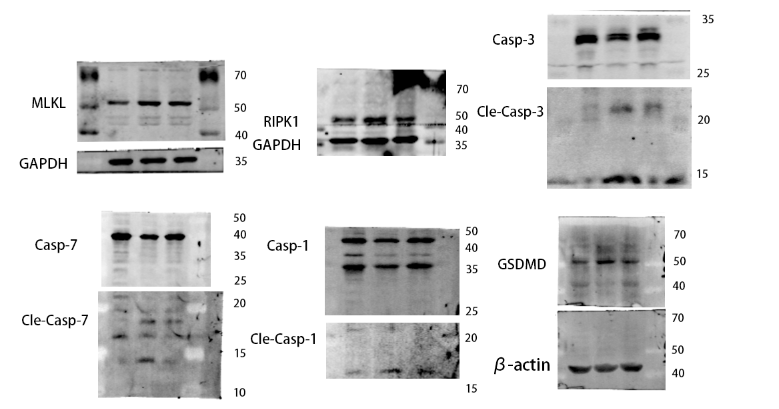


**Repeat 3**


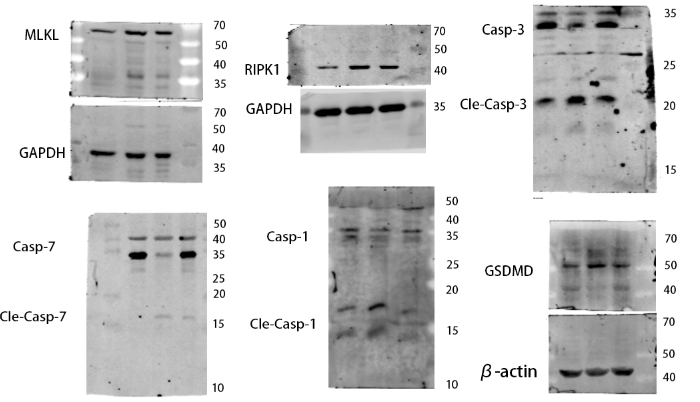


**Figure 4 A**

**Repeat 1**


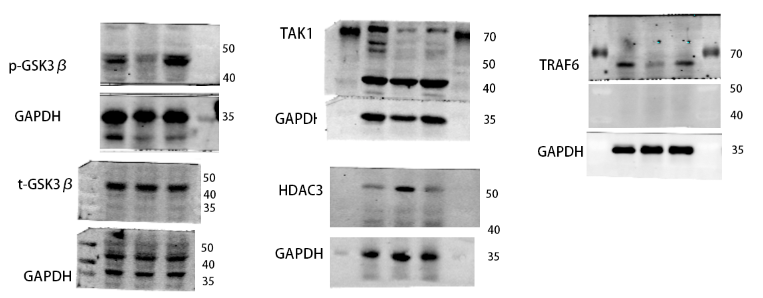


**Repeat 2**


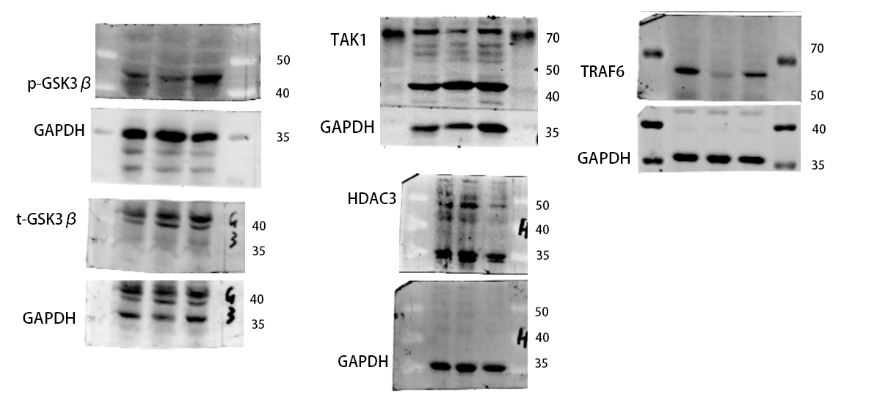


**Repeat 3**


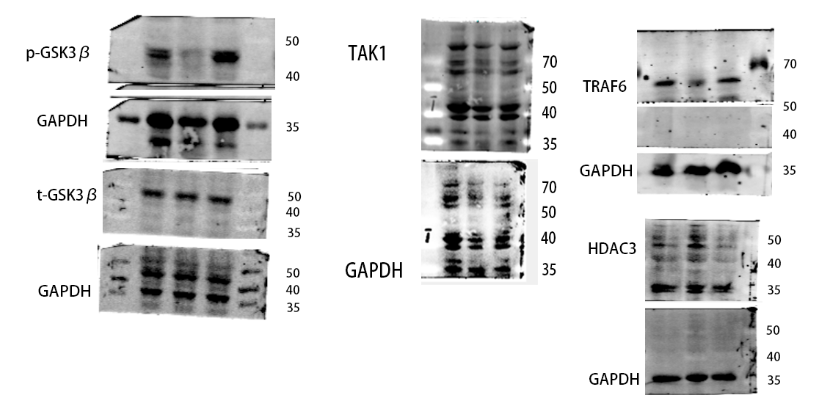


**Figure 4 D**

**Repeat 1**


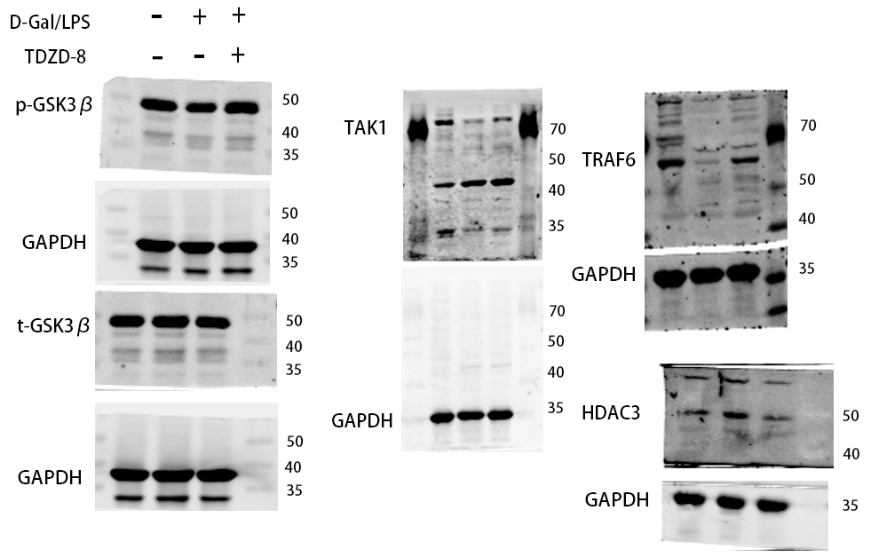


**Repeat 2**


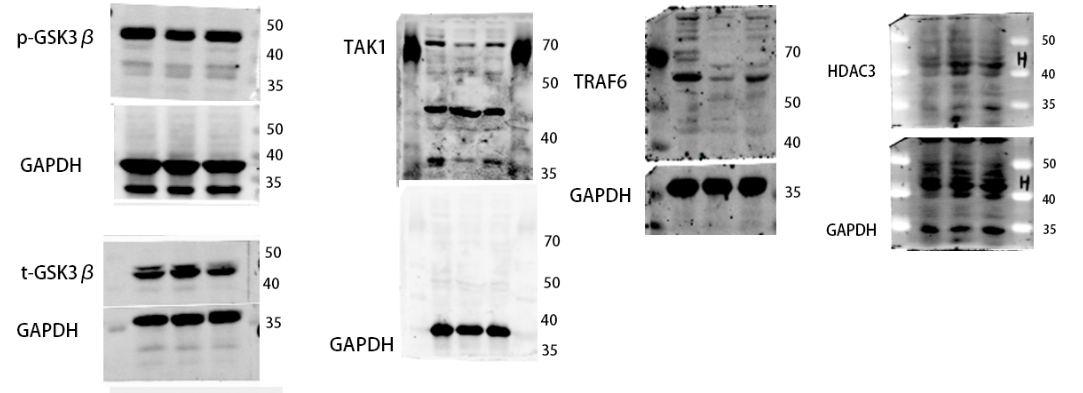


**Repeat 3**


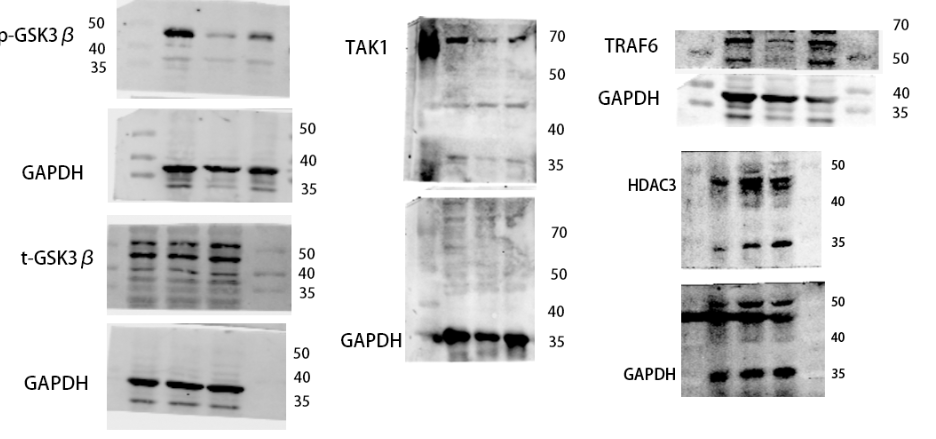


**Figure 5 E**

**Repeat 1**


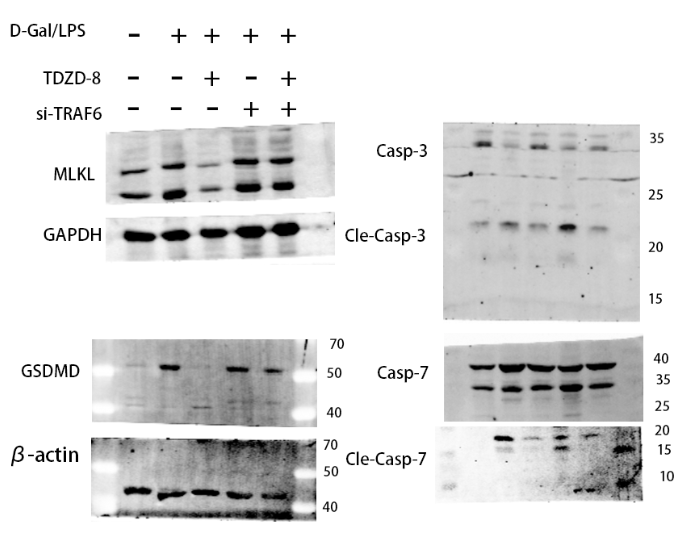


**Repeat 2**


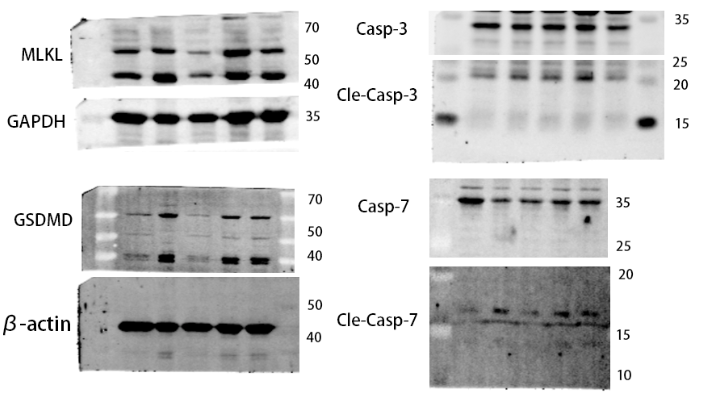


**Repeat 3**


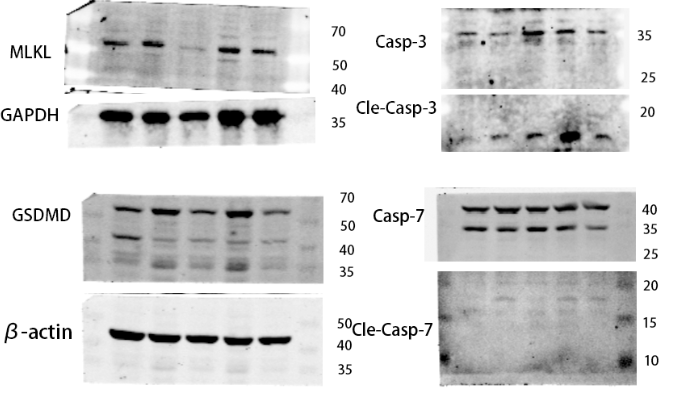


**Figure 6 A**

**Repeat 1**


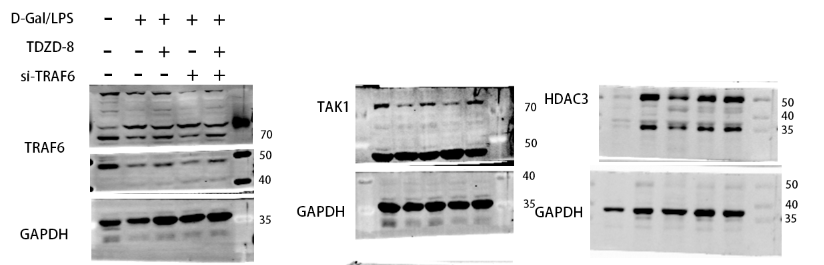


**Repeat 2**


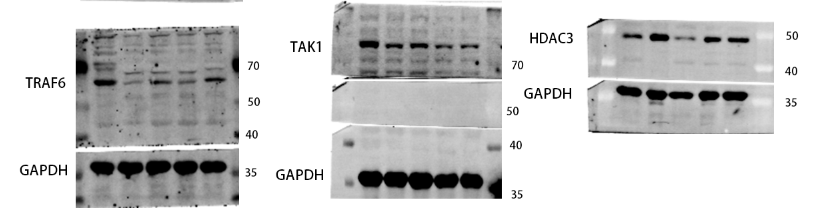


**Repeat 3**


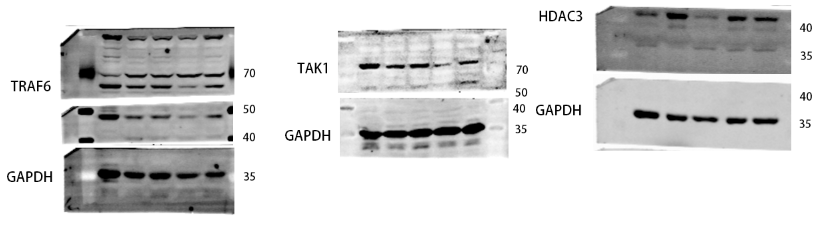


**Figure 6 E**


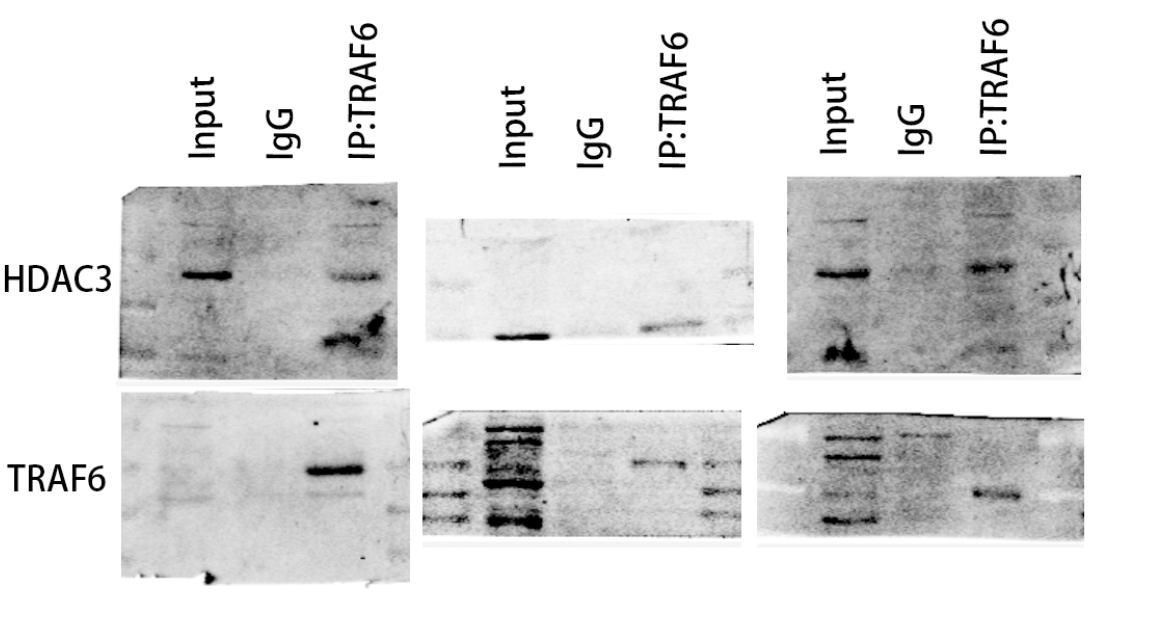


**Figure 7 C**


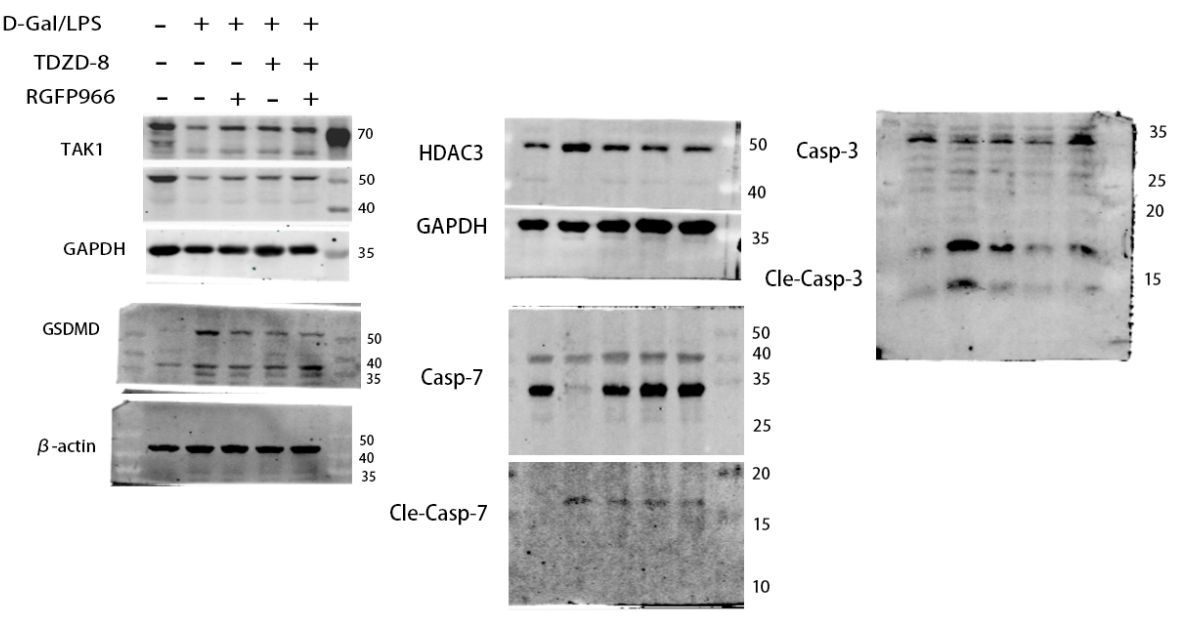


**Repeat 2**


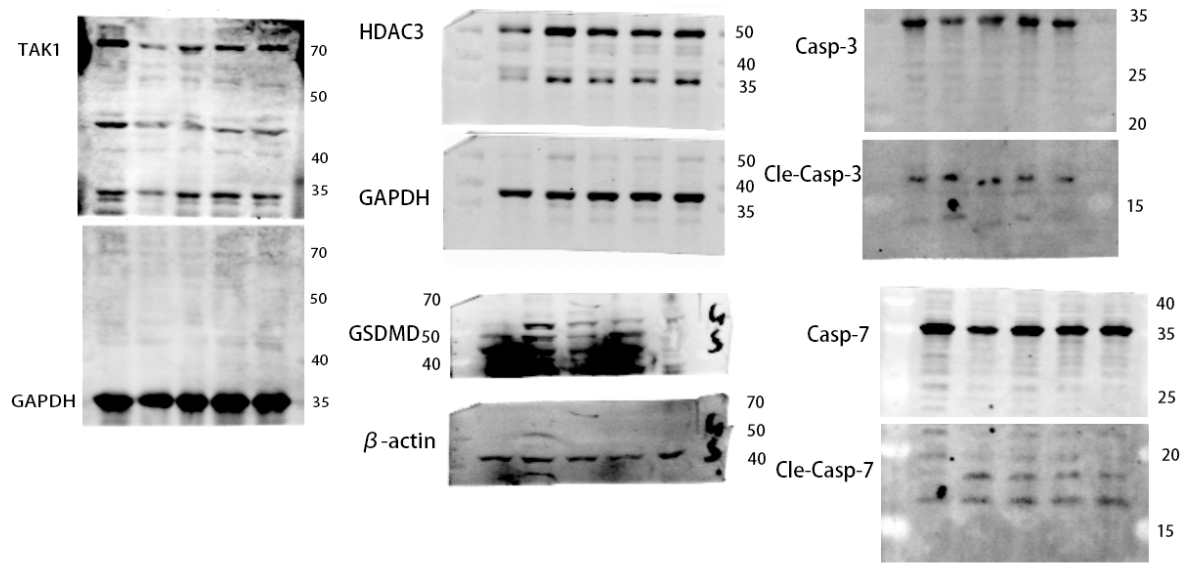


**Repeat 3**


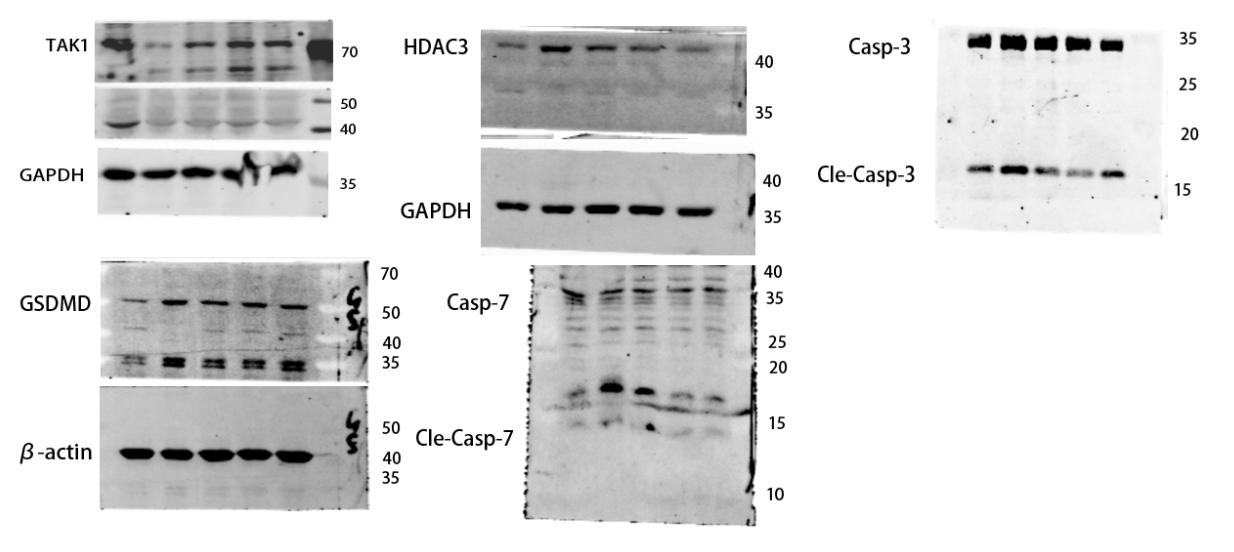

Supplement: Supplementary file 1 — Additional file 1. [file 12950_2023_350_MOESM1_ESM.docx]
